# Complete mitochondrial genome of *Micractinium singularis* MM0003 (Chlorellaceae, Trebouxiophyceae)

**DOI:** 10.1080/23802359.2020.1715896

**Published:** 2020-01-24

**Authors:** Seung-Woo Jo, Nam Seon Kang, Hyunsik Chae, Jung A Lee, Kyeong Mi Kim, Moongeun Yoon, Ji Won Hong, Ho-Sung Yoon

**Affiliations:** a Department of Energy Science, Kyungpook National University, Daegu, Korea;; b School of Life Sciences, Kyungpook National University, Daegu, Korea;; c Department of Taxonomy and Systematics, National Marine Biodiversity Institute of Korea, Seocheon, Korea

**Keywords:** Complete mitochondrial genome, *Micractinium singularis*, Chlorellaceae, Trebouxiophyceae

## Abstract

The mitochondrial genome of *Micractinium singularis* MM0003 was completely sequenced. This mitogenome has 75,931 bp in length and consists of 62 genes including 32 protein-coding, 3 rRNA, and 27 tRNA genes. The overall GC content of the genome is 27.5%.

*Micractinium* Fresenius is a ubiquitous genus commonly found in various aquatic environments. *Micractinium singularis* H. Chae, H.-G. Choi & J. H. Kim (Chlorellaceae, Trebouxiophyceae), a new species of the genus, was recently described by Chae et al. (2019). *Micractinium singularis* KSF0094, isolated from Deception Island, South Shetland Islands, Antarctica, is the holotype of the species. It is considered that *M*. *singularis* and its close relatives possess psychrotolerant properties. In this study, the complete mitogenome of *M*. *singularis* MM0003 is reported for the first time.

*Micractinium singularis* MM0003 was isolated from Janghang Harbor, Seocheon, Korea (36°00′23.96″N 126°41′ 23.52″E). Morphological characteristics and molecular phylogenetic evidences inferred from the small subunit 18S rRNA and internal transcribed spacer sequence data indicated that strain MM0003 belonged to this newly described species. This strain was deposited at the National Marine Biodiversity Institute of Korea (MABIK) and Korean Collection for Type Cultures (KCTC) under the accession numbers of MABIK-LP-00000134 and KCTC 13290BP, respectively. The culture was grown in BG-11 medium (UTEX, Austin, TX, USA) at 18 °C under cool fluorescent light (approximately 40 µmole m^−2^ s^−1^) in a light:dark cycle (14:10 hrs) for 4 weeks until growth was apparent. Biomass was harvested by centrifugation at 2,063 ×*g* (1580 R; Labogene, Daejeon, Korea). Whole genomic DNA was extracted from the sample using a DNeasy Plant Mini Kit (Qiagen, Hilden, Germany) followed by the preparation of a library using an MGIEasy DNA Library Prep Kit V1 (BGI, Shenzhen, China) according to the manufacturer’s instruction. Whole genome sequencing was performed using BGISEQ-500 (BGI, China) sequencer and raw data were filtered to obtain >10 Gb clean data per each sample. *De novo* mitogenome assembly was carried out using NOVOPlasty v3.6 software (Dierckxsens et al. 2017). The size of the circular mitogenome produced is 75,931 bp (GenBank accession number MN894286) which is slightly larger than those of the previously reported *M*. *pusillum* (70,061 bp, GenBank accession number MN649871) and *M*. *conductrix* (74,708 bp, GenBank accession number KY629619) mitogenomes. The nucleotide composition is 36.7% A, 35.7% T, 13.6% G, and 13.9% C. The overall GC content is 27.5%. The *M*. *singularis* mitogenome contains 62 genes, including 32 predicted protein-coding, 3 rRNA, and 27 tRNA genes. All 32 genes were revealed as complete protein-coding genes, which all of them were started with ATG as a start codon and ended by a stop codon (30 genes by TAA, 1 gene by TAG, and 1 gene by TGA). It was found that none of the genes were overlapped and 27 tRNA genes ranged from 49 to 86 bp in length. Phylogenetic analysis was performed by using PhyML 3.0 with 12 reported mitogenome sequences (Fan et al. 2017) belonging to the Trebouxiophyceae family and the phylogenetic tree was visualized by FigTree v1.4.4. The result displayed the phylogenetic position of strain MM0003 within Trebouxiophyceae (Figure 1). This new sequence information would contribute to a better understanding of the phylogenetic relationships of the *Micractinium* species and mitochondrial genome diversity and evolution in the family Trebouxiophyceae.

**Figure 1.**
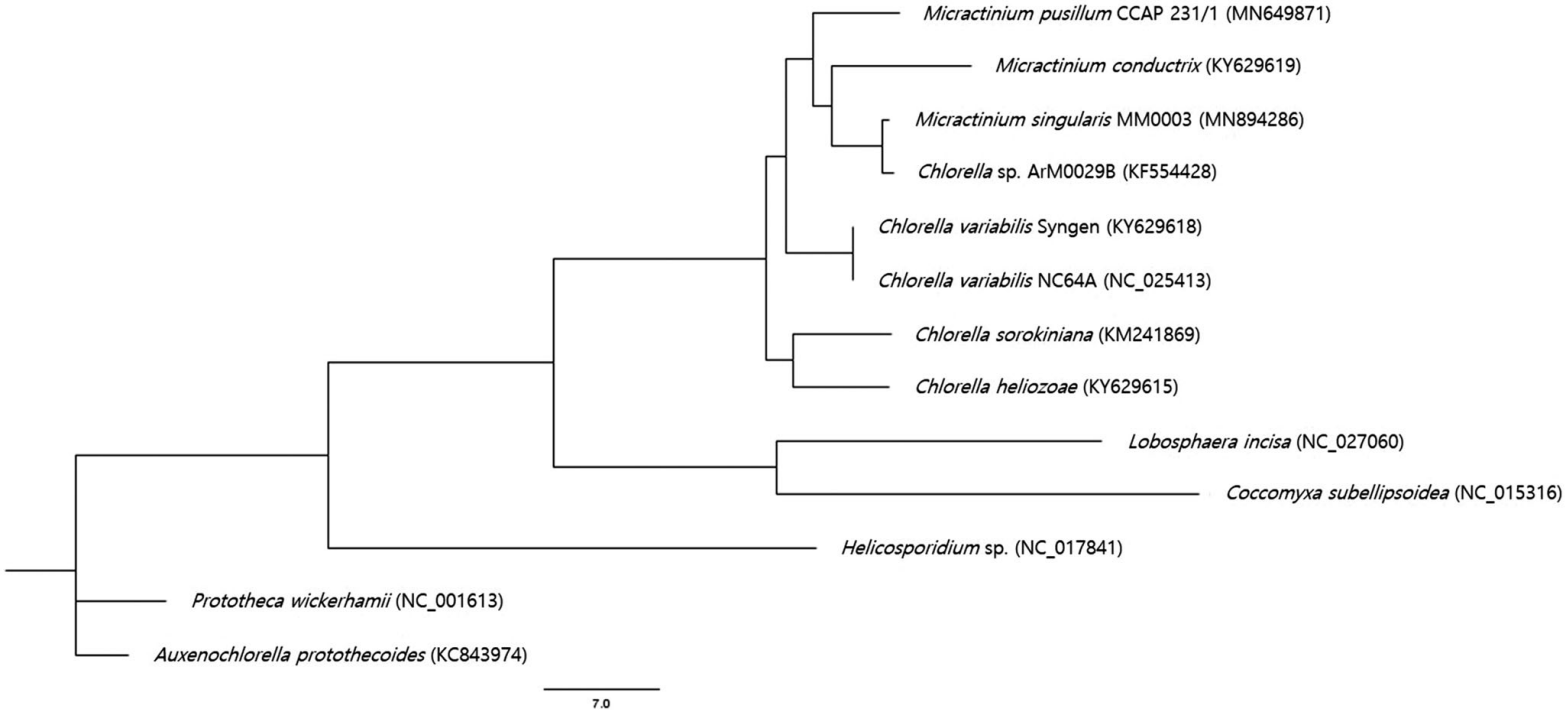
Maximum-likelihood phylogenetic tree of *M. singularis* MM0003 and 12 other species. GenBank accession numbers were indicated in the parentheses.
